# Deaths Related to Domestic Violence in Washington State

**DOI:** 10.1001/jamanetworkopen.2024.29974

**Published:** 2024-09-04

**Authors:** Julie M. Kafka, Avanti Adhia, David D. Martin, Ayah Mustafa, Ali Rowhani-Rahbar, Frederick P. Rivara

**Affiliations:** 1 Firearm Injury & Policy Research Program, University of Washington, Seattle; 2 Department of Pediatrics, School of Medicine, University of Washington, Seattle; 3 School of Nursing, University of Washington, Seattle; 4 King County Prosecuting Attorney’s Office, Seattle, Washington; 5 Department of Epidemiology, University of Washington, Seattle

## Abstract

**Importance:**

Domestic violence (DV; including intimate partner and family violence) is associated with heightened lethality risks, yet limited research has comprehensively assessed the connection between DV and fatal violence considering both homicides and suicides. Understanding the fatal consequences of DV can point to missed opportunities to support individuals and their families.

**Objective:**

To assess the proportion of violent deaths that were connected to DV and describe contacts with the legal system or social services prior to each DV-related fatality.

**Design, Setting, and Participants:**

This cross-sectional study used quantitative and qualitative data from the National Violent Death Reporting System (NVDRS) for all individuals who died by homicide or suicide in Washington from January 1, 2015, to December 31, 2020. Analyses were conducted from August 1, 2022, to September 30, 2023.

**Main Outcomes and Measures:**

A multipronged approach was used to assess DV history using existing NVDRS variables, leveraging data from prior review of NVDRS death narratives, applying a validated natural language processing tool, and linking related deaths. Domestic violence was recorded as yes or no, but the decedent’s role in the abusive relationship (ie, experiencing or enacting DV) could not be differentiated. To describe system involvement prior to each death, keyword searching and hand review of NVDRS death narratives were used.

**Results:**

A total of 7352 intentional violent deaths (1192 homicides [16.2%]; 6160 suicides [83.8%]) with known circumstances were recorded in Washington during the study period. Of these, 948 deaths (12.9%) were connected to DV (624 [65.8%] among males; mean [SD] age at death, 45.3 [19.2] years), including 588 suicides (62.0%) and 360 homicides (38.0%). For 420 DV-related deaths (44.3%), there was evidence to suggest that the person who died or their intimate partner(s), family, or cohabitants had prior contacts with the legal system or social services. Specifically, 318 records (33.5%) mentioned prior contacts with law enforcement or the criminal legal system (eg, prior 9-1-1 calls, criminal convictions), and 225 (23.7%) described engagement with social services or the civil legal system (eg, civil protection order, divorce, or child custody problems).

**Conclusions and Relevance:**

In this cross-sectional study, 12.9% of violent deaths in Washington were connected to DV. The findings suggest that more resources are needed to support law enforcement, court professionals, and social services specialists to proactively identify and refer families to wraparound supports before the situation can escalate to a fatality.

## Key Points

**Question:**

What were the proportions of homicides and suicides connected to domestic violence (DV) in Washington from 2015 to 2020, and what were the missed opportunities for prevention?

**Findings:**

In this cross-sectional study of 7352 intentional violent deaths in Washington from 2015 to 2020, 13% had known DV histories. For 44% of DV-related fatalities, prior contacts with social services or the legal system were documented, with 9-1-1 calls and civil protection orders being the most common.

**Meaning:**

These findings suggest that surveillance of DV-related fatalities can help identify potential opportunities to interrupt escalating violence, including using 9-1-1 calls and civil protection orders, for intervention and referral efforts.

## Teaser

This cross-sectional study evaluates the proportion of homicides and suicides connected to domestic violence in Washington and contacts with the legal system or social services before these deaths.

## Introduction

Domestic violence (DV) is a pervasive problem in the US. In 2022, there were over 1400 telephone calls daily to the national DV hotline.^1^ Domestic violence includes any physical violence, sexual violence, psychological abuse, or stalking by a family member or a current or former intimate partner. This includes various DV subtypes, such as spousal violence, dating violence, child abuse, and in-law abuse.^2,3,4^ Intimate partner violence (IPV) is the most common form of DV,^5^ but DV subtypes often co-occur and involve other individuals.^6,7^ According to a 2019 meta-analysis, when one form of violence is present in the home, there is greater odds of another type of DV occurring (odds ratio, 6.0; 95% CI, 1.5-21.5).^8^

Domestic violence may be associated with negative outcomes for all involved parties, including emotional distress, mental health problems, physical injury, and even death.^9^ In the US, 1 in 5 homicides and over half of homicides with female decedents are perpetrated by a current or former intimate partner or close family member.^10,11,12,13^ Others can also be killed; in the IPV context, over 20% of homicide decedents may be outside the primary abusive relationship (eg, children, new dating partners, or other family members).^7^

Individuals who are involved in DV may also die by suicide.^14^ For example, an adult experiencing IPV may fatally attempt suicide after losing custody of a shared child to their abusive partner, or a person who has perpetrated DV may die by suicide after being charged with sexual abuse of a child in the family.^14,15,16^ Researchers have documented an association between DV and nonfatal suicidality^17,18,19^ and between DV and homicide-suicides^20,21,22,23^ but the association between DV and isolated suicide events (ie, not connected to homicide) is often overlooked.^24^

Understanding the fatal consequences of DV, including both homicide and suicide, can provide insights into missed opportunities to offer support and interventions for individuals and families.^25^ In prior research, Kafka et al^25^ considered the contribution of IPV to fatal violence in the US and found that IPV was associated with at least 16.3% of homicides and 7.1% of suicides. These national estimates, however, are incomplete; because IPV falls under the legal definition for DV,^2,3,4^ some death data mention DV broadly without specifying the subtype of violence.^14^ This would cause unspecified DV-related deaths to be discounted in prior work and family violence–related deaths not to be considered at all.^25^

For the present study, we focused on all DV-related violent deaths, including those that were related to IPV, child abuse, or other family violence. Services to address DV are often integrated,^1^ and thus there may be common opportunities to intervene. Building on existing research,^26,27,28^ we also documented whether any involved party had prior contacts with social services, law enforcement, and/or the legal system prior to the death. Our objective was to comprehensively enumerate DV-related fatalities and precursors to those deaths to help distinguish opportunities to identify and interrupt escalating violence in Washington, where our study was located.

## Methods

### Data Source

For this cross-sectional study, we used data from the National Violent Death Reporting System (NVDRS), which is coordinated by the Centers for Disease Control and Prevention and administered at the state level. After a violent death has occurred, trained abstractors from each funded state review death certificates and death investigation reports from the coroner or medical examiner and law enforcement. Based on these secondary data, NVDRS abstractors record demographics of the decedent (eg, age, sex), incident characteristics (eg, relationship between the suspect and decedent), and known circumstances for each death (eg, IPV, other abuse). Circumstance variables are recorded in a binary manner as present or absent (ie, not present, not available, or unknown).^29^ The NVDRS abstractors also compile text summaries of the coroner or medical examiner and law enforcement reports. With both quantitative and qualitative data, the NVDRS is considered the most comprehensive data source for fatal violence in the US.^30^ Still, the NVDRS is limited based on the details available in secondary data reports, most of which come from coroner or medical examiner and law enforcement death investigations.^29^ Based on institutional guidelines from the University of Washington, this study was considered nonhuman participant research, given the use of administrative data from deceased individuals. We followed Strengthening the Reporting of Observational Studies in Epidemiology (STROBE) reporting guideline for cross-sectional studies.

This study was prompted by a request from local partners in Washington to provide information on DV-related fatalities to inform monitoring and prevention. In-depth research about the precursors to fatal violence is often only possible at the state level; opportunities to intervene may be state specific, and in-depth hand review of NVDRS text data often is not feasible nationally. Thus, we examined NVDRS data from Washington from January 1, 2015, to December 31, 2020. Washington used a phased implementation for NVDRS data collection, capturing 60% to 80% of violent deaths from 2015 to 2017 and 100% thereafter. Washington has similar rates of violent death compared with the US overall, although suicide rates were slightly higher in Washington in 2020 (15.2 per 100 000 people vs 13.4 per 100 000 people in the US overall) and homicide rates were lower (4.2 per 100 000 people in vs 7.7 per 100 000 people in the US overall).^31,32^ We examined all homicides and suicides for which circumstance information was available in the NVDRS, as is best practice.^33^

### Measures

#### Domestic Violence

We documented whether DV was described as a contributing factor for each death (yes or no), including IPV, child abuse, or other family violence. We used a multipronged approach (Figure). Domestic violence was documented as yes (ie, present) regardless of whether the decedent was described as an individual who experienced DV, perpetrated DV, or was a corollary party, as these details are sometimes unclear or unavailable.^14^

**Figure.**
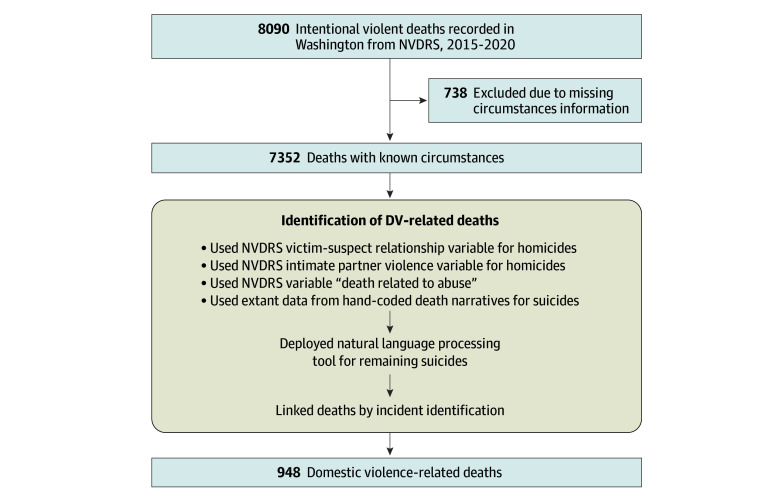
Inclusion Criteria and Identification Process for Domestic Violence (DV)–Related Fatalities NVDRS indicates National Violent Death Reporting System.

First, we used NVDRS variables for IPV, abuse or neglect by a caregiver, and the decedent-suspect relationship to identify DV cases.^29^ Qualifying relationships included current or former spouse, dating partner, or other family member (eg, grandparent, biological child). We also included cases in which the decedent was family or an intimate partner of a current or former intimate partner to capture corollary homicides (eg, child of suspect’s girlfriend, boyfriend of decedent’s mother).

The aforementioned variables were recorded systematically for homicides but not for suicides.^29^ For suicides, we leveraged existing hand-coded death narratives (n = 214), from which we conducted multistate projects to identify IPV-related suicides.^14,26,28^ Intimate partner violence is a subset of DV; thus, any case coded as IPV was considered DV. Subsequently, we deployed a natural language processing (NLP) tool for remaining suicide cases to identify mentions of DV in the death narrative texts.^26^ The NLP tool was designed to identify IPV circumstances by looking for keywords or phrases related to IPV, such as sexual violence, physical injury of an intimate partner, coercive control, and fear by or of an intimate partner.^26^ It then uses a random forest approach to categorize each death as IPV related (yes or no; accuracy, 0.96; sensitivity, 0.70; specificity, 0.98; positive predictive value, 0.72).^26^ It may also be useful for detecting other DV; many narratives of cases near the decisional boundary for IPV described other family violence or only mentioned DV broadly,^26^ thus meeting our DV case definition for the current study. We performed quality checks to confirm acceptable performance (eAppendix 1 and eFigure in Supplement 1) before adopting the tool’s assigned yes or no labels as the DV designation.

Finally, we linked deaths by incident identifier. If DV was noted for any death in a multideath incident (eg, homicide-suicide, multiple homicide), all fatalities were considered DV related. Any death labeled as a mercy killing was coded as not related to DV.

#### Prior System Involvement

We examined whether the decedent, the suspect, their intimate partner(s), family, or cohabitants were described in the NVDRS death narratives as having contacts with social services or the legal system prior to the fatality. We only considered contacts that occurred a day or more prior to the death to focus on proactive opportunities for intervention. To construct these measures, we used keyword searching and hand coding (eAppendix 2 in Supplement 1).

We identified prior civil legal or social service system involvement, which included any divorce proceedings, child custody problems, civil domestic violence protection order (DVPO) cases, or engagement with Child Protective Services. Separately, we identified prior criminal legal system involvement, which included prior incarceration (including mention of probation), convictions (eg, having a criminal record), charges (eg, upcoming court dates), arrests, ongoing investigations (including mentions of a warrant or detainment), or other indications that the parties were known to law enforcement (eg, prior 9-1-1 calls to the residence). We reported how common these interactions were overall and the overlap in prior contacts across systems.

#### Other Variables

We adopted NVDRS language for decedent biological sex and race and ethnicity.^29^ Race and ethnicity categories included American Indian or Alaska Native, Asian or Pacific Islander, Black, Latino or Hispanic, White, and another or unknown race. Due to small cell counts, non-Hispanic decedents whose race information was “other” or “unknown” were presented in a single category. Race and ethnicity were included in the study to allow an assessment of possible racialized disparities in DV-related fatalities. Legal intervention deaths (shootings by active-duty law enforcement) were shown as a subtype of homicide given small cell counts.

Existing NVDRS variables capture some information about prior system contacts, but these measures are not necessarily comprehensive.^29^ To contrast our in-depth text review, we also presented information on these existing NVDRS variables about civil and criminal legal problems.

### Statistical Analysis

Analyses were conducted from August 1, 2022, to September 30, 2023. We presented raw counts stratified by death manner. Small cell counts (n < 10) were suppressed in accordance with reporting requirements of the Centers for Disease Control and Prevention. We conducted a sensitivity analysis excluding 2020 data to observe whether findings changed outside the COVID-19 pandemic. All data cleaning and analyses were conducted in R, version 4.2.3 (R Program for Statistical Computing; March 15, 2023).

## Results

A total of 7352 intentional violent deaths (1192 homicides [16.2%]; 6160 suicides [83.8%]) with known circumstances were recorded in Washington during the study period (Figure). Of these deaths, 948 (12.9%) were DV-related violent deaths. Among the DV-related homicides and suicides, 324 (34.2%) were female and 624 (65.8%) were male; mean (SD) age at death was 45.3 (19.2) years. In terms of race and ethnicity, 21 (2.2%) were American Indian or Alaska Native; 49 (5.2%), Asian or Pacific Islander; 55 (5.8%), Black; 108 (11.4%), Latino or Hispanic; 673 (71.0%), White; and 42 (4.4%), another or unknown racial group (Table 1).

**Table 1.** Characteristics of Intentional Violent Deaths in Washington From 2015 to 2020

Characteristic	Deaths, No. (%) (N = 7352)^a^			
	Homicide		Suicide	
	All (n = 1192)	DV related (n = 360)	All (n = 6160)	DV related (n = 588)
**Demographics**				
Sex				
Female	312 (26.2)	204 (56.7)	1459 (23.7)	120 (20.4)
Male	880 (73.8)	156 (43.3)	4701 (76.3)	468 (79.6)
Age, y				
<10	51 (4.3)	47 (13.1)	S	S
10-24	252 (21.1)	45 (12.5)	899 (14.6)	54 (9.2)
25-40	443 (37.2)	111 (30.8)	1594 (25.9)	252 (42.9)
41-54	259 (21.7)	83 (23.1)	1416 (23.0)	165 (28.1)
55-70	137 (11.5)	49 (13.6)	1503 (24.4)	92 (15.6)
≥71	50 (4.2)	25 (6.9)	744 (12.1)	25 (4.3)
Race and ethnicity				
American Indian or Alaska Native	46 (3.9)	9 (2.5)	103 (1.7)	12 (2.0)
Asian or Pacific Islander	74 (6.2)	20 (5.6)	332 (5.4)	29 (4.9)
Black	223 (18.7)	33 (9.2)	160 (2.6)	22 (3.7)
Latino or Hispanic	215 (18.0)	52 (14.4)	393 (6.4)	56 (9.5)
White	575 (48.2)	222 (61.7)	4956 (80.5)	451 (76.7)
Other or unknown race^b^	59 (4.9)	24 (6.7)	216 (3.5)	18 (3.1)
Marital status				
Single or never married	718 (60.2)	171 (47.5)	2336 (37.9)	144 (24.5)
Married	218 (18.3)	101 (28.1)	1750 (28.4)	256 (43.5)
Divorced or separated	212 (17.8)	77 (21.4)	1645 (26.7)	177 (30.1)
Other or unknown	44 (3.7)	S	429 (7.0)	S
Educational attainment				
Less than high school	365 (30.6)	116 (32.2)	888 (14.4)	81 (13.8)
High school degree	493 (41.4)	116 (32.2)	2163 (35.1)	228 (38.8)
More than high school	321 (26.9)	125 (34.7)	3019 (49.0)	275 (46.8)
Unknown	13 (1.1)	S	90 (1.5)	S
Decedent served in the military	70 (5.9)	16 (4.4)	1201 (19.5)	115 (19.6)
**Incidents**				
Type				
Isolated	991 (83.1)	245 (68.1)	6051 (98.2)	514 (87.4)
Homicide-suicide	94 (7.9)	77 (21.4)	88 (1.4)	72 (12.2)
Multiple deaths or other^c^	107 (9.0)	38 (10.6)	21 (0.3)	S
Primary weapon or lethal means				
Firearm	828 (69.5)	193 (53.6)	2945 (47.8)	364 (61.9)
Strangulation or hanging	28 (2.3)	15 (4.2)	1738 (28.2)	133 (22.6)
Poisoning	S	S	874 (14.2)	51 (8.7)
Sharp instrument	155 (13.0)	68 (18.9)	156 (2.5)	11 (1.9)
Other	177 (14.8)	82 (22.8)	447 (7.3)	29 (4.9)
Legal intervention death^d^	157 (13.2)	21 (5.8)	NA	NA

Abbreviations: DV, domestic violence; NA, not applicable or not routinely collected; S, suppressed.

^a^ Cell counts less than 10 or cells that revealed information about counts less than 10 were suppressed in accordance with Centers for Disease Control and Prevention guidelines. Percentages have been rounded and may not total 100.

^b^ Information on other racial groups was not available. Non-Hispanic individuals who were multiracial or with unknown race were presented together due to small cell counts.

^c^ Included events that had multiple homicides, multiple suicides, or a combination of homicides and legal intervention deaths.

^d^ Decedent was fatally injured by law enforcement officer in the line of duty. These deaths are sometimes reported separately from homicides, but they were combined here due to small cell counts.

Among DV-related fatalities, 189 (19.9%) occurred as part of an incident in which multiple people died. Firearms were the most common weapon used in DV-related fatalities (557 [58.7%]). Suicides were the most common type of DV-related fatality (588 [62.0%]); 360 fatalities (38.0%) were homicides. Most DV-related suicides (514 [87.4%]) were isolated events (ie, not homicide-suicide or multideath events).

Circumstances of DV were most common among females who died by homicide; 204 of 312 females who died by homicide (65.4%) died in connection to DV compared with 156 of 880 males who died by homicide (17.7%). Among suicides, proportions with DV circumstances were similar for males (468 of 4701 [10.0%]) and females (120 of 1459 [8.2%]). The largest number of DV-related fatalities occurred among males who died by suicide (468 [49.4%]).

When children (age <10 years) were killed in a homicide, most of the time (47 of 51 [92.2%]) that homicide was connected to DV. Older adult homicide decedents (age ≥70 years) were also commonly killed in connection to DV (25 of 50 [50.0%]).

### DV-Related Fatalities and Prior System Involvement

Our in-depth hand review of text narratives focused solely on DV-related fatalities (n = 948) (Table 2). For 225 DV-related deaths (23.7%), we found mention of prior contacts with the civil legal system or social services in NVDRS death narratives. These included 74 DV-related fatalities (7.8%) among individuals who had been impacted by divorce, 40 (4.2%) among those who had been dealing with child custody problems, 128 (13.5%) with narratives that mentioned a DVPO, and 31 (3.3%) among individuals who had been involved with Child Protective Services. For 318 DV-related deaths (33.5%), there was prior contact with the criminal legal system, including incarceration (64 [6.8%]), conviction (37 [3.9%]), charges (41 [4.3%]), arrests (62 [6.5%]), criminal investigations (33 [3.5%]), or prior 9-1-1 calls to the residence (182 [19.2%]).

**Table 2.** Prior System Involvement for Decedents, Suspects, Intimate Partners, Family Members, or Cohabitating Residents Among DV-Related Fatalities in Washington From 2015 to 2020

System involvement	DV-related deaths, No. (%)^a^		
	All (n = 948)	Homicide (n = 360)	Suicide (n = 588)
Civil legal or social services^b^	225 (23.7)	84 (23.3)	141 (24.0)
Divorce	74 (7.8)	22 (6.1)	52 (8.8)
Child custody problems	40 (4.2)	20 (5.6)	20 (3.4)
Civil protective order	128 (13.5)	41 (11.4)	87 (14.8)
Involvement with Child Protective Services	31 (3.3)	17 (4.7)	14 (2.4)
Prior contact with law enforcement and the criminal legal system^b^	318 (33.5)	142 (39.4)	176 (29.9)
Incarceration	64 (6.8)	31 (8.6)	33 (5.6)
Conviction	37 (3.9)	25 (6.9)	12 (2.0)
Charges or court date	41 (4.3)	S	S
Arrest	62 (6.5)	19 (5.3)	43 (7.3)
Investigation or warrant	33 (3.5)	15 (4.2)	18 (3.1)
Prior contacts (9-1-1 calls)	182 (19.2)	109 (30.3)	73 (12.4)
Patterns of system involvement			
Civil legal or social services only	102 (10.8)	30 (8.3)	72 (12.2)
Law enforcement or criminal legal system only	195 (20.6)	88 (24.4)	107 (18.2)
Contacts with both	123 (13.0)	54 (15.0)	69 (11.7)
Contacts with neither	528 (55.7)	188 (52.2)	340 (57.8)
Existing NVDRS variables^c^			
Civil legal problems	NA	NA	140 (23.8)
Criminal legal problems	NA	NA	74 (12.6)

Abbreviations: DV, domestic violence; NA, not applicable or not routinely collected; NVDRS, National Violent Death Reporting System; S, suppressed.

^a^ Cell counts less than 10 or cells that revealed information about counts less than 10 were suppressed in accordance with Centers for Disease Control and Prevention guidelines.

^b^ Initiated any time before the day of the fatal incident based on any mention in the death narratives. Subcategories were not mutually exclusive.

^c^ Whether civil or criminal legal problems contributed to suicide. Only cases for which the circumstance was recorded as present are shown. This information was not systematically documented for homicides. There was no specified time frame in the NVDRS coding manual for these variables. These categories are not mutually exclusive. Unlike the hand-coded measures, the NVDRS measures can include crimes or civil or criminal legal proceedings that began on the day of the fatality.

Patterns across these systems revealed that 102 DV-related deaths (10.8%) had only civil legal or social service involvement, 193 (20.4%) had only criminal legal system involvement, 123 (13.0%) had contacts across both settings, and 528 (55.7%) had no contacts documented with either (a total of 420 [44.3%] with prior contact). The hand review revealed substantially more instances of prior civil or criminal legal system contacts compared with the conventional NVDRS indicators used to capture this information (Table 2).

### Sensitivity Analysis

When data from 2020 were excluded, the same proportion of DV-related fatalities overall was found (803 of 6236 [12.9%]). Similar patterns of prior systems engagement were found among DV-related fatalities (82 [10.2%] civil only; 155 [19.3%] criminal or law enforcement only; 102 [12.7%] both; 464 [57.8%] neither).

## Discussion

This cross-sectional study found that DV was associated with 12.9% of intentional violent deaths in Washington from 2015 to 2020. This estimate is higher than that reported in prior research focused only on IPV, in which IPV was documented in 9.5% of violent deaths nationally.^27^ We also found that among DV-related deaths, 44.3% of narratives mentioned prior contact with social services or the legal system. Findings suggest that there may be multiple opportunities to identify and interrupt DV before it can escalate to fatality.

Calls to 9-1-1 and DVPOs were the most reported type of system involvement prior to a DV-related fatality. These may be among the most common avenues through which survivors and/or concerned family members formally ask for help.^34^ Thus, 9-1-1 calls and DVPOs could be considered key settings to identify, refer, and follow up with families, including by connecting them to wraparound services, such as DV shelters, family justice centers, or mental health counseling services. Often, a constellation of survivor-centered supports are necessary to adequately address DV.^35,36^ These referrals, however, do not always happen; both the civil legal system and law enforcement officers are often underresourced to address DV.^37,38^ Availability of a DV advocate in the DVPO process or the use of a co-responder model during 9-1-1 calls could help facilitate referrals.^39^ Interventions may be particularly important when children are also in the home, as we found that 92.2% of children younger than 10 years who died by homicide were killed in connection to DV. Qualitative research and community-engaged work can help inform tailored intervention and referral processes.

Consistent with research by our group on IPV-related fatalities,^27^ the present findings highlight the importance of considering homicides in addition to suicides when conceptualizing DV-related fatalities. We found a larger number of DV-related suicides compared with DV-related homicides, although DV was more common among homicides than among suicides. Domestic violence fatality reviews and researchers focused on DV should consider broadening their scope of work to assess the role of DV in suicide.^24^

Domestic violence–related fatalities are gendered.^40,41^ A larger proportion of females who died by homicide died in connection to DV compared with males who died by homicide, while proportions of DV were similar by sex for suicides. Domestic violence experiences and fatality risks among men have historically been overlooked, and given the large number of DV-related suicides among males, additional research in this area is warranted.

Most DV-related deaths had firearms as the lethal mechanism. Federal law already prohibits individuals convicted of felony crimes or DV misdemeanors and those subject to DVPOs from purchasing or possessing firearms.^42^ These federal prohibitions, however, are not adequately implemented or enforced.^43^ In 2017, King County, Washington, introduced an innovative regional unit to help identify and remove dangerous weapons (including firearms) from prohibited individuals. Since implementation of these efforts, King County has documented increased weapon relinquishment by prohibited persons.^44^ This may be an important approach to prevent DV-related fatalities. Extreme risk protection orders can also facilitate the removal of firearms, a process that is already available in Washington, 20 other states, and the District of Columbia.^45,46^ Ensuring that the public knows about DVPOs and extreme risk protection orders and that individuals are supported during the petitioning process should be a priority for states.

Findings also point to other state-level intervention possibilities. Having a comprehensive statutory definition for DV allows nonphysical abuse to be recognized.^47^ Using integrated data systems to clearly track DV cases may also help ensure a smooth handoff across agencies. Ultimately, allocating more funding for survivor support services (eg, shelters), evidence-based interventions to address IPV perpetration, and mental health treatment programs can help individuals better manage acute lethality risks.^48,49,50^

We call for more efforts to assess the role of DV in both homicide and suicide with a focus on opportunities for prevention. This can include consideration of screening and referral practices during routine interactions with the legal system and for social services related to DV. In particular, 9-1-1 calls and DVPO petitioning may represent valuable, missed opportunities to de-escalate DV and connect families to supportive, wraparound services. In addition, there appears to be an urgent need to address the safety and well-being of children in the home who are not only at risk for fatality themselves but who also may lose parents and caregivers to DV. Finally, improved data infrastructure to monitor and study the role of DV in fatalities can facilitate more holistic, comprehensive research on effective and tailored interventions.^51^

### Limitations

This study has some limitations. A total of 55.7% of DV-related deaths in the sample did not include textual descriptions of prior system contacts. Either involved parties were not interfacing with these systems or these interactions were not documented in NVDRS. The NVDRS text summaries are primarily compiled from coroner or medical examiner and law enforcement reports, which do not always include comprehensive information.^52,53^ Given limitations with underreporting, our findings should be considered conservative estimates.

Homicides and suicides are investigated differently.^54^ Even within the same death manner, NVDRS narratives may contain more or less detail based on decedent characteristics (ie, among suicides, narratives contain more information when the decedent is older, male, and belongs to certain racial groups).^52^ Differential recording practices could have led to systematic measurement error in our data.

NVDRS data only included a subset of all fatalities in Washington from 2015 to 2017, mostly drawn from larger counties during this early period. Findings may be skewed to represent data from urban areas, and results may not generalize to other states. Still, prior research suggests similar rates of IPV-related fatalities nationally,^25,27,28^ which bolsters our confidence in the generalizability of these results.

We took a holistic approach to collectively examine DV-related fatalities, including deaths related to IPV, child abuse, or other family violence. We were not able to investigate differences among these subtypes due to small cell counts and measurement limitations, nor were we able to systematically identify the decedent’s role in DV. This is an important area for future research.

In addition, we used a case-only sample of individuals who died. We could not make inferences from these data about fatality risks in living populations.

## Conclusions

In this cross-sectional study, 12.9% of violent deaths in Washington were connected to DV. For 44.3% of DV-related fatalities, prior contacts with social services or the legal system were documented, with 9-1-1 calls and civil protection orders being most common. The findings suggest that more resources are needed to support law enforcement, court professionals, and social services specialists to proactively identify and refer families to wraparound supports before DV can escalate to a fatality.

## Notes

Supplement 1. **eAppendix 1.** Assessing of the Utility of the NLP Tool for Detecting DV-Related Suicides **eFigure.** Error Rate Among Hand-Reviewed Cases (n = 365) Near the Yes/No Decisional Boundary for Mention of Domestic Violence Circumstances, 2018-2020 **eAppendix 2.** Additional Details About Operationalization of Variables
eReferences.

Supplement 2.
Data Sharing Statement
