# Supplementary material for: Deaths Related to Domestic Violence in Washington State
Source: JAMA Netw Open. 2024 Sep 4;7(9):e2429974. doi: 10.1001/jamanetworkopen.2024.29974 (PMC11375474; doi:10.1001/jamanetworkopen.2024.29974)
Supplement: Supplement 1. — eAppendix 1. Assessing of the Utility of the NLP Tool for Detecting DV-Related Suicides eFigure. Error Rate Among Hand-Reviewed Cases (n = 365) Near the Yes/No Decisional Boundary for Mention of Domestic Violence Circumstances, 2018-2020 eAppendix 2. Additional Details About Operationalization of Variables eReferences. [file jamanetwopen-e2429974-s001.pdf]

Supplementary Online Content

Kafka JM, Adhia A, Martin DD, Mustafa A, Rowhani-Rahbar A, Rivara FP. Deaths related to domestic violence in Washington State. *JAMA Netw Open*. 2024;7(8):e2429974. doi:10.1001/jamanetworkopen.2024.29974

eAppendix 1. Assessing of the Utility of the NLP Tool for Detecting DV-Related Suicides

eFigure. Error Rate Among Hand-Reviewed Cases (n=365) Near the Yes/No Decisional Boundary for Mention of Domestic Violence Circumstances, 2018-2020

eAppendix 2. Additional Details About Operationalization of Variables

eReferences.

This supplementary material has been provided by the authors to give readers additional information about their work.

eAppendix 1. Assessing of the Utility of the NLP Tool for Detecting DV-Related Suicides

A Natural Language Processing (NLP) tool was developed and validated to detect mentions of intimate partner violence (IPV) circumstances in suicide death narratives for the National Violent Death Reporting System (NVDRS).[1] We sought to confirm whether it was appropriate to use this tool for detecting domestic violence (DV) circumstances. DV is a broader type of violence including IPV as well as other family violence (e.g., between a parent and child or cohabitating roommates). Preliminary research suggested that the NLP tool may be capable of detecting both IPV and broader mentions of DV.[1]

To confirm whether this NLP tool could be used in the present study, we deployed the NLP tool and subsequently conducted hand-review for a sub-sample of cases to confirm whether the death was indeed associated with DV or not. We conducted this quality checking for 2018-2020 cases that were near the NLP tool's decisional boundary for a yes/no label (i.e., cases that received an IPV score close to 0.5). By taking this approach, we effectively quality checked cases that the tool was most uncertain about (e.g., cases that were "probably" or "maybe" IPV/DV). Specifically, cases were reviewed that received an IPV score between 0.3-0.7 (i.e., 0.5 +/- 0.2)

In total, n=365 suicide cases were hand-reviewed.[1] We determined that n=243 (66.6%) of these "high uncertainty" cases had been categorized correctly by the NLP tool. We corrected n=45 false positives and n=77 false negatives. The human-corrected labels were adopted for the main study.

Results from our quality checking are further described in **eFigure 1**. As expected, the error rate was highest among cases that received a IPV score closest to the decisional boundary (score of 0.5, indicating high uncertainty). The false positive rate dropped precipitously as the NLP tool's IPV score increased, suggesting that cases with high IPV scores truly did have IPV or DV circumstances

---

[1] Cases prior to 2018 were not hand-reviewed given limited time and resources

© 2024 Kafka JM et al. *JAMA Network Open*.

described in the suicide death narratives. Cases with IPV scores below 0.5 would be labelled by the NLP tool as no IPV. Among these cases, the false negative rate decreased more gradually, likely because any DV-related suicide that mentioned general family violence without explicit mention of IPV would originally have been labeled as “no” in the gold standard that the tool was trained on, and thus may be missed in the present application. Still, results from our quality checking exercise demonstrated that the NLP tool could be used to identify DV circumstances for suicide, particularly when paired by human hand-review of high-uncertainty cases. It is important to note, however, that overall accuracy, sensitivity, precision, or positive predictive value of the NLP tool in the context of the present project were not calculated given our purposive sampling of cases for quality checks.

eFigure. Error Rate Among Hand-Reviewed Cases (n=365) Near the Yes/No Decisional Boundary for Mention of Domestic Violence Circumstances, 2018-2020

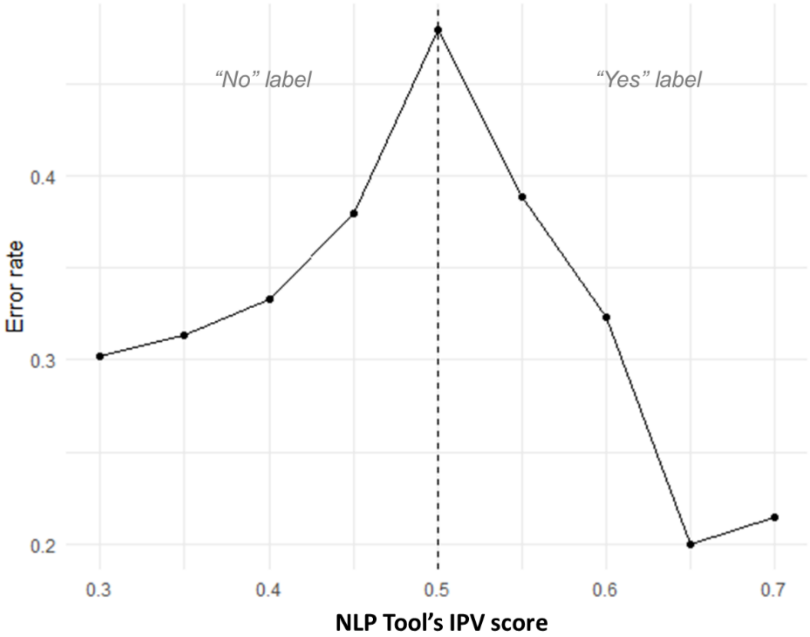

NLP, Natural Language Processing; IPV, intimate partner violence; DV, domestic violence

Note: The decisional boundary (an IPV score of 0.5) is shown as a vertical, dashed line.

© 2024 Kafka JM et al. *JAMA Network Open*.

eAppendix 2. Additional Details About Operationalization of Variables

To hand-code variables in the study related to prior involvement with social services or the civil legal system, keywords or phrases were first identified for each variable of interest. We also considered common misspellings and syntactical variations for each keyword and phrase (e.g., protection order; protective order; order protecting). Any sentences from the death narrative text that contained those keywords or phrases was subsequently hand-reviewed by the first author, who then labeled that variable as present (i.e., yes) or absent (i.e., no). Sometimes, the whole narratives were reviewed to provide additional context.

A similar but slightly modified process was used for coding prior involvement with law enforcement or the criminal justice system; threats to law enforcement; or decedent occupation as a law enforcement officer. All approached are further described below.

Legal divorce: We searched for any text that contained the word “divorce.” We only counted cases as divorce-related if the death narrative suggested that the parties had legally initiated or recently completed the divorce process. If a case merely mentioned that there was ongoing “separation”, “estrangement”, or the decedent and their partner were considering divorce, then we recorded divorce as no.

Child custody: We performed keyword searching for mentions of “custody.” All returned text was hand-reviewed to confirm whether there indeed had been a prior or active child custody cases. We considered mention of “child support” or “visitation” with a biological child in the narratives to be a custody issue. If child custody was discussed only in relation to actions taken subsequent to the violent fatality, custody would be recorded as no.

Civil protective order cases. To identify textual mentions of civil protective orders, we searched for the following two-word phrases: “protection order,” "restraining order," "no contact

© 2024 Kafka JM et al. *JAMA Network Open*.

order." We also reviewed any case where the word "order" occurred in the same sentence with either: "protect," "restrain," "contact," or "violation." Finally, we considered text that included the acronyms, "pfa," "ppo," "po," "dvpo," "dvro," "epo,"or "tpo." Requests for a civil protection order were coded as yes, even if that request was later dropped.

Child protective services (CPS). We searched for textual mentions of "child protective services," "CPS," or instances where the word "child" and "protect" occurred in the same sentence. If contact with CPS was mentioned only as a subsequent action that occurred resulting from a DV-related fatality, then the case was coded as no.

Prior involvement with law enforcement or the criminal justice system. If any of the following NVDRS variables were endorsed, we coded the case as yes: victim known to authorities; suspect contact with police; or suspect recently released (from jail, prison, or a detention facility).

We also hand-reviewed death narratives for all cases where the variable flagging recent criminal legal problems had been endorsed. This variable can capture instances when the decedent was involved in an ongoing criminal legal court case, but it also can capture scenarios where the decedent was committing a crime on the same day as the fatal event, even when that crime was not previously known to law enforcement.[2] In the latter scenario, the death would have been coded as no; our measure was designed only to capture involvement with law enforcement or the criminal legal system that occurred at least a day or more prior to the fatality.

As a final strategy, we also examined cases that mentioned any of these keywords: "charge," "arrest", "crime," "criminal," "court," "trail," "jail," "prison," "incarceration," "jury," "felony," "misdemeanor," "conviction," "warrant," "apprehend," "violation," and "offense."

© 2024 Kafka JM et al. *JAMA Network Open*.

A second coder assisted with coding this variable. The two coders together reviewed 50 cases of returned text to ensure they were both labelling consistently. Subsequently, 10% (n=50) of new (unseen) cases were double-coded. Only three disagreements were identified, and discrepancies were resolved through consensus. All other cases were independently reviewed.

After we had determined whether the case should be coded for prior involvement with law enforcement or the criminal justice system, the first author characterized the type of involvement (e.g., prior 911 calls, arrest, formerly incarcerated) by reviewing the extracted text that coders had deemed as relevant for their labelling decision.

© 2024 Kafka JM et al. *JAMA Network Open*.

eReferences.

1. Kafka JM, Fliss MD, Trangenstein PJ, Reyes LM, Pence BW, Moracco KE. Detecting intimate partner violence circumstance for suicide: development and validation of a tool using natural language processing and supervised machine learning in the National Violent Death Reporting System. *Injury Prevention*. 2022;doi:10.1136/ip-2022-044662

2. Center for Disease Control and Prevention. *National Violent Death Reporting System (NVDRS) Web Coding Manual, Version 5.5*. 2021. www.cdc.gov/injury

© 2024 Kafka JM et al. *JAMA Network Open*.
