# Supplementary material for: Deaths Related to Domestic Violence in Washington State
Source: JAMA Netw Open. 2024 Sep 4;7(9):e2429974. doi: 10.1001/jamanetworkopen.2024.29974 (PMC11375474; doi:10.1001/jamanetworkopen.2024.29974)
Supplement: Supplement 2. — Data Sharing Statement [file jamanetwopen-e2429974-s002.pdf]

Data Sharing Statement

Kafka. Deaths Related to Domestic Violence in Washington State. *JAMA Netw Open*. Published August 23, 2024. doi:10.1001/jamanetworkopen.2024.29974

Data
Data available: No

Additional Information
Explanation for why data not available: Data belong to a third party provider. These data are available upon request from the WA State Department of Health.
